# Culture independent assessment of human milk microbial community in lactational mastitis

**DOI:** 10.1038/s41598-017-08451-7

**Published:** 2017-08-10

**Authors:** Shriram H. Patel, Yati H. Vaidya, Reena J. Patel, Ramesh J. Pandit, Chaitanya G. Joshi, Anju P. Kunjadiya

**Affiliations:** 1 Ashok and Rita Patel Institute of Integrated Study and Research in Biotechnology and Allied Sciences, ADIT campus, New Vallabh Vidyanagar, Anand, Gujarat India; 2 Department of Animal Biotechnology, College of Veterinary Science and Animal Husbandry, Anand Agricultural University, Anand, Gujarat India; 3 Center for Interdisciplinary Studies in Science and Technology (CISST), Sardar Patel University, Vallabh Vidya Nagar, Gujarat India

## Abstract

Breastfeeding undoubtedly provides important benefits to the mother-infant dyad and should be encouraged. Mastitis, one of the common but major cause of premature weaning among lactating women, is an inflammation of connective tissue within the mammary gland. This study reports the influence of mastitis on human milk microbiota by utilizing 16 S rRNA gene sequencing approach. We sampled and sequenced microbiome from 50 human milk samples, including 16 subacute mastitis (SAM), 16 acute mastitis (AM) and 18 healthy-controls. Compared to controls, SAM and AM microbiota were quite distinct and drastically reduced. Genera including, *Aeromonas*, *Staphylococcus*, *Ralstonia*, *Klebsiella*, *Serratia*, *Enterococcus* and *Pseudomonas* were significantly enriched in SAM and AM samples, while *Acinetobacter*, *Ruminococcus*, *Clostridium*, *Faecalibacterium* and *Eubacterium* were consistently depleted. Further analysis of our samples revealed positive aerotolerant odds ratio, indicating dramatic depletion of obligate anaerobes and enrichment of aerotolerant bacteria during the course of mastitis. In addition, predicted functional metagenomics identified several gene pathways related to bacterial proliferation and colonization (e.g. two-component system, bacterial secretion system and motility proteins) in SAM and AM samples. In conclusion, our study confirmed previous hypothesis that mastitis women have lower microbial diversity, increased abundance of opportunistic pathogens and depletion of commensal obligate anaerobes.

## Introduction

Breastfeeding gives a unique opportunity for improving infant health, at the same time, maternal health^1^. Breast milk is recommended as sole source of nutrition for rapidly growing infant, and reduces the risk of infection and illness^2–4^. Gut microbiome acquired during breastfeeding also protects the newborn from pathogenic bacteria and contributes to the development and maturation of infant immune system^5, 6^. Several studies have reported that human milk is a rich source of many mutualistic, beneficial and potentially probiotic bacteria that may confer immunity to newborn infant^7^. Recently, by utilizing Next Generation Sequencing (NGS) technology, healthy human milk microbiome has been analyzed for bacterial diversity and stability in different geographical regions^8–14^.

Mastitis is an inflammation of mammary gland, during which dysbiosis of human milk microbiome exhibits outgrow of opportunistic pathogenic bacteria and reduction of healthy commensal bacteria^15, 16^. Different population-based studies conducted in USA^17^, New Zealand^18^, Finland^19^ and Australia^20^ revealed that 20–25% of breastfeeding women are at the risk of developing mastitis during lactation period. Even though mastitis is a very common and distressing condition among lactating women, studies dealing with human lactational mastitis are scarce. We and others have previously demonstrated higher abundance of *Staphylococcus aureus* and *Staphylococcus epidermidis*, a species considered as common colonizer of skin and mucosa, together with bacteria belonging to the genus *Streptococci*, *Corynebacterium*, *Pseudomonas*, *Klebsiella* and *Enterococcus* form mastitis samples^15, 21–23^.

Recent development of culture-independent tool has dramatically improved our understanding of unculturables and its functioning^24^. Healthy human milk microbiome has been studied in relation to gestation time, birthing mode and infant gender in diverse populations^11^. However, only one study has explored microbial dysbiosis associated with lactational mastitis through metagenomic approach. Jimenez *et al*. 2015, carried out metagenomic shotgun sequencing of breast milk sample collected from 10 women with mastitis (5 from SAM and 5 from AM) and 10 healthy-controls. Similarly to the results obtained from culture dependent studies, *Staphylococcus* (*S*. *aureus* in acute and *S*. *epidermidis* in subacute mastitis) and *Pseudomonas* were the predominant genus in subacute and acute mastitis cases. Moreover, they found that SAM and AM women had decreased microbial diversity and higher sequences related to presumptive etiological agents compared to control^16^.

In this context, we first time report influence of SAM and AM on human milk microbiome through amplicon sequencing approach. We show that women having SAM and AM had distinct microbial community profile, reduced microbial diversity and higher abundance of opportunistic pathogens compared to healthy-controls.

## Results

### Study population

We enrolled 50 women, including 16 SAM, 16 AM and 18 healthy-controls for the study. Characteristic of the study population (age and days postpartum) is shown in Supplementary Table S1. Attending physician at different Primary Health Care (PHC) and Community Health Care (CHC) center of Valsad and Vadodara region of Gujarat, India had diagnosed them based on local sign and symptoms of mastitis. Sixteen women were confirmed to have SAM (breast engorgement and fever), whereas other sixteen women were reported to be suffering from AM (pain, redness and swelling on breast). Eighteen age-matched healthy women also donated human milk sample as a control.

### Sequencing summary

A total of 50 human milk samples, collected from healthy, SAM and AM women were sequenced in the V2-V4 region of 16S rRNA gene, resulting in 3.44 million raw reads (Supplementary Table 2). Initial quality filtering generated 2.14 million high quality sequences, corresponding to 30.94% of usable sequences for healthy-control, 27.94% for SAM and 41.11% for AM samples, which were taxonomically annotated using MG-RAST server (Supplementary Table S2). Subsequently, high quality sequences were downloaded from MG-RAST server and subjected to QIIME v1.9 analysis pipeline for alpha and beta diversity analysis. Reads were clustered into 6203 Operational Taxonomic Units (OTU) at 97% sequence similarity with a mean average of 1148 ± 409 per sample.

### Human milk microbiota associated with mastitis changes at the phylum level

Human milk meta-community classified into known 25 phyla, 185 family and 590 genera. *Proteobacteria* (49.63%) and *Firmicutes* (16.62%) were the two most dominant bacterial phyla, whereas *Actinobacteria*, *Spirochaetes*, *Synergistetes*, *Tenericutes* and *Bacteroidetes* constituted minor phyla, each contributing more than 0.1% of total sequences (Table 1). Uncultured bacteria accounted for 36.73%, 34.89% and 22.32% of total bacterial sequences in healthy-controls, SAM and AM samples, respectively. SAM and AM samples were largely enriched in *Proteobacteria*, while *Firmicutes* and *Actinobacteria* contributed to higher proportion of healthy bacterial sequences. On comparison, it was found that, *Firmicutes* were significantly differed between healthy-acute (non-parametric Mann-Whitney test, P < 0.001) and acute-subacute mastitis samples (non-parametric Mann-Whitney test, P < 0.005).

**Table 1:** Proportion of bacterial phyla detected in breast milk of healthy, SAM and AM women.

Phylum	Healthy (Mean ± SD)	Subacute mastitis (Mean ± SD)	Acute mastitis (Mean ± SD)
*Proteobacteria*	41.14 ± 25.92	55.41 ± 21.47	52.34 ± 28.89
unclassified bacteria	36.73 ± 25.36	22.32 ± 23.49	34.89 ± 27.59
*Firmicutes*	18.33 ± 10.45	21.57 ± 14.42	9.96 ± 18.54
*Actinobacteria*	1.54 ± 0.86	0.20 ± 0.16	0.89 ± 0.76
*Bacteroidetes*	0.52 ± 0.38	0.128 ± 0.125	0.53 ± 0.71
*Spirochaetes*	0.01 ± 0.012	0.13 ± 0.30	0.79 ± 2.18
*Synergistetes*	0.82 ± 0.55	0.007 ± 0.011	0.012 ± 0.16
*Tenericutes*	0.53 ± 0.44	0.05 ± 0.121	0.10 ± 0.12

### Microbial richness and diversity decreases during mastitis

**Figure 1:**
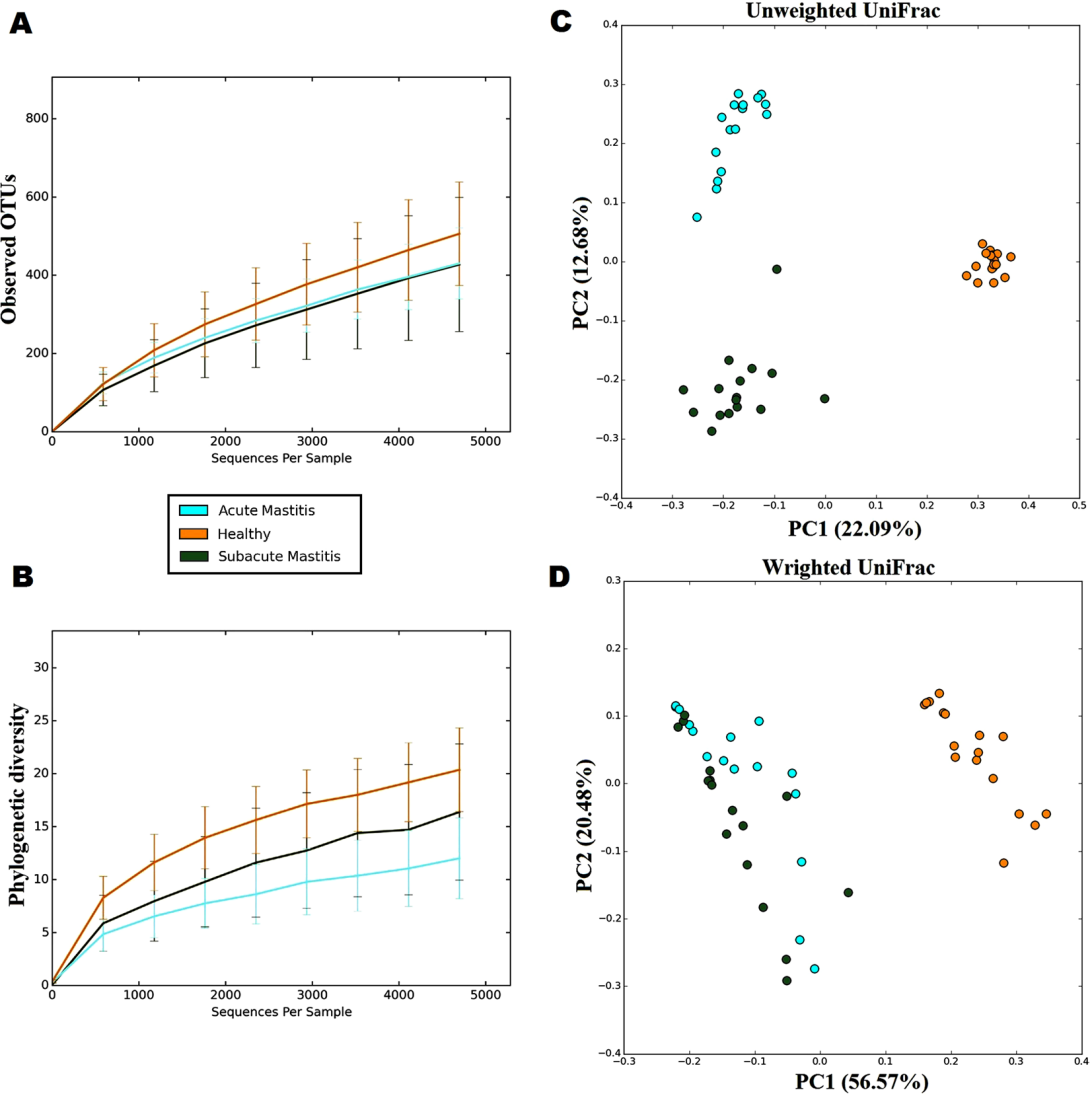
Alpha diversity and beta diversity of breast milk microbiota among healthy-controls, SAM and AM women. Alpha diversity was determined by (**A**) number of observed OTUs and (**B**) Phylogenetic diversity. Beta diversity was accessed by using PCoA of (**C**) unweighted UniFrac and (**D**) weighted UniFrac distance metrices. Clear separation was observed between healthy-controls, SAM and AM samples.

We then compared species richness, estimated by number of observed OTUs and biodiversity accessed by Phylogenetic Diversity (PD) among the three groups of participants. OTU table was rarefied to 4718 sequences to limit the effect of different sequencing depth per sample. Using non-parametric Kruskal-Wallis test for comparison, we found that healthy-controls had significantly higher Phylogenetic Diversity compared to SAM and AM samples (Fig. 1B; P < 0.001). However, there was decreasing trend in number observed OTUs in SAM and AM individuals, it did not reach statistical significant (Fig. 1A; Non-parametric Kruskal Wallis test, P = 0.137). We next determined inter-sample variability in community structure by calculating difference in beta diversity using unweighted and weighted UniFrac distance metrics. We observed significant difference in beta diversity as measure by unweighted UniFrac PCoA (Principle Coordinate Analysis) among three group of participants (Fig. 1C; adonis statistics: R^2^–0.33; P = 0.001 and anosim statistics: R - 0.91; P = 0.001). Similarly, weighted UniFrac PCoA displayed substantial variation in overall microbial community structure (Fig. 1D; adonis statistics: R^2^–0.91; P = 0.001 and anosim statistics: R - 0.70; P = 0.001). In addition, PCoA plotted based on Bray-Curtis distance matrix was used to examine differences and similarity at all taxonomic level (from phylum to species). Although, we could not observed any separation between healthy, SAM and AM samples at phylum level, clustering was more pronounced at family, genus and species (OTU) level taxa (Supplementary Fig. 1). Moreover, hierarchical clustering of OTUs based on ward linkage clustering method also revealed three different clusters of SAM and AM and healthy-controls (Supplementary Fig. 2).

### Microbial dysbiosis in subacute and acute mastitis

**Figure 2:**
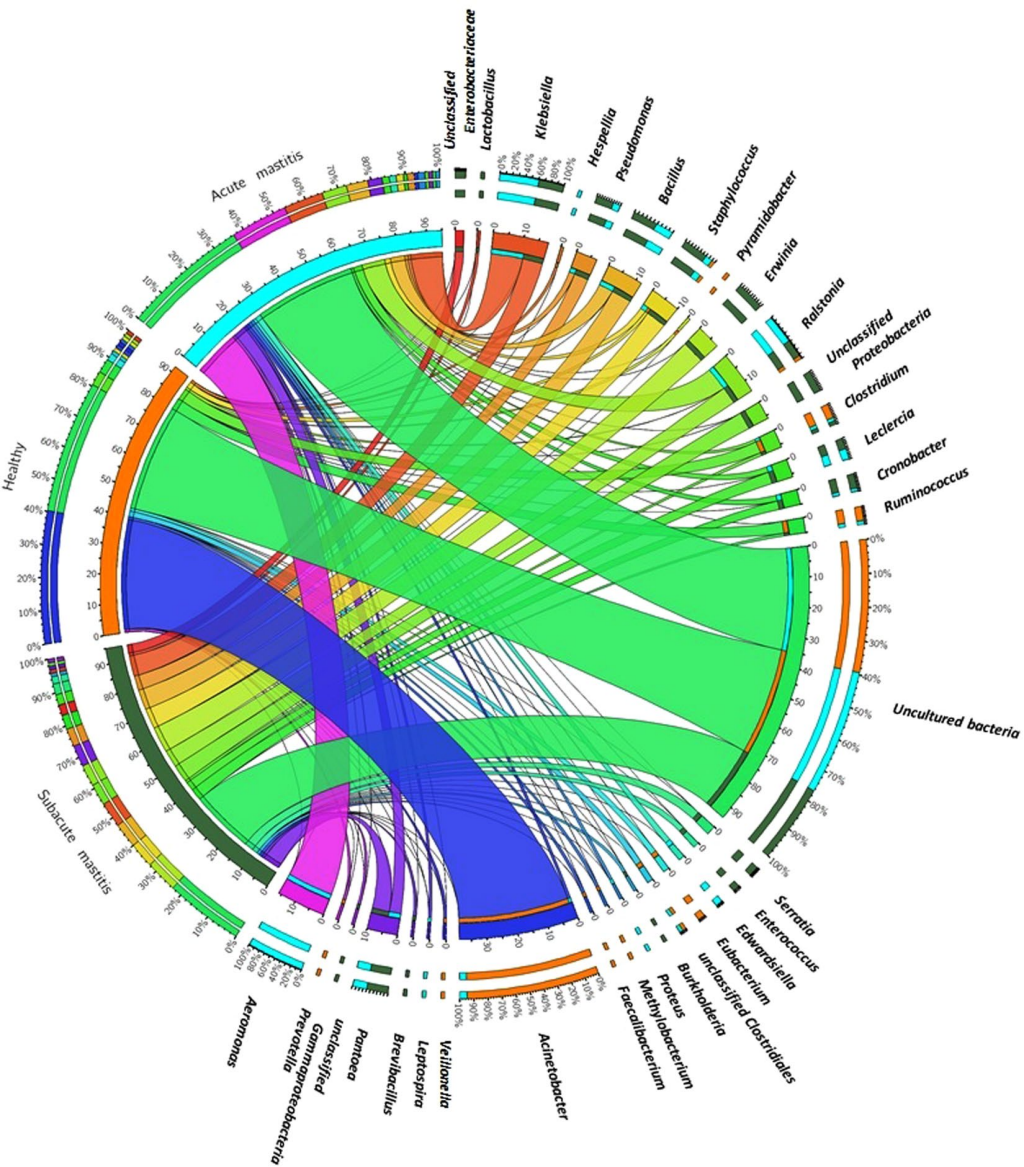
Circos representation of top most abundant bacterial genera between healthy-controls, SAM and AM samples. Bacterial genera having abundance above 0.2% of all bacterial sequences were plotted.

Distinct bacterial communities were detected in healthy-controls, SAM and AM mastitis, which tend to differed in both composition and abundance. We identified a total of 590 different genus in all the individuals, including 395 genus in healthy-controls, 343 genus in SAM cases and 559 genus in AM cases. At genus level, healthy-controls possessed relatively more *Acinetobacter* (37.08%), *Ruminococcus* (4.08%), *Clostridium* (4.31%) and *Eubacterium* (1.51%) in comparison to other groups. Whereas, AM and SAM mastitis milk microbiota were enriched in *Aeromonas* (15.97% in AM vs 0.005% in SAM), *Staphylococcus* (1.94% vs 7.25%), *Klebsiella* (10.99% vs 6.84%), *Ralstonia* (7.56% vs 5.90%), *Bacillus* (5.11% vs 6.84%), *Pantoea* (4.21% vs 5.77%), *Serratia* (0.036% vs 2.58%), *Enterococcus* (0.057% vs 1.93%) and *Pseudomonas* (1.57% vs 5.21%). Moreover, we observed that a group of 13 bacterial genera (*Acinetobacter*, *Bacillus*, *Clostridia*, *Staphylococcus*, *Lactobacillus*, *Propionibacterium*, *Corynebacterium*, *Pseudomonas*, *Paenibacillus*, *Prevotella*, *Ralstonia*, *Eubacterium* and *Ruminococcus*), frequently identified in previous milk metagenomics study, were detected in at least 47 out of 49 individuals tested, constituting the core microbiome. Genus with more than 1% of total sequences were defined as predominant and these predominant genera contributed 87.38%, 91.14% and 90.66% of total sequences in healthy-controls, SAM and AM individuals, respectively (Fig. 2).

**Figure 3:**
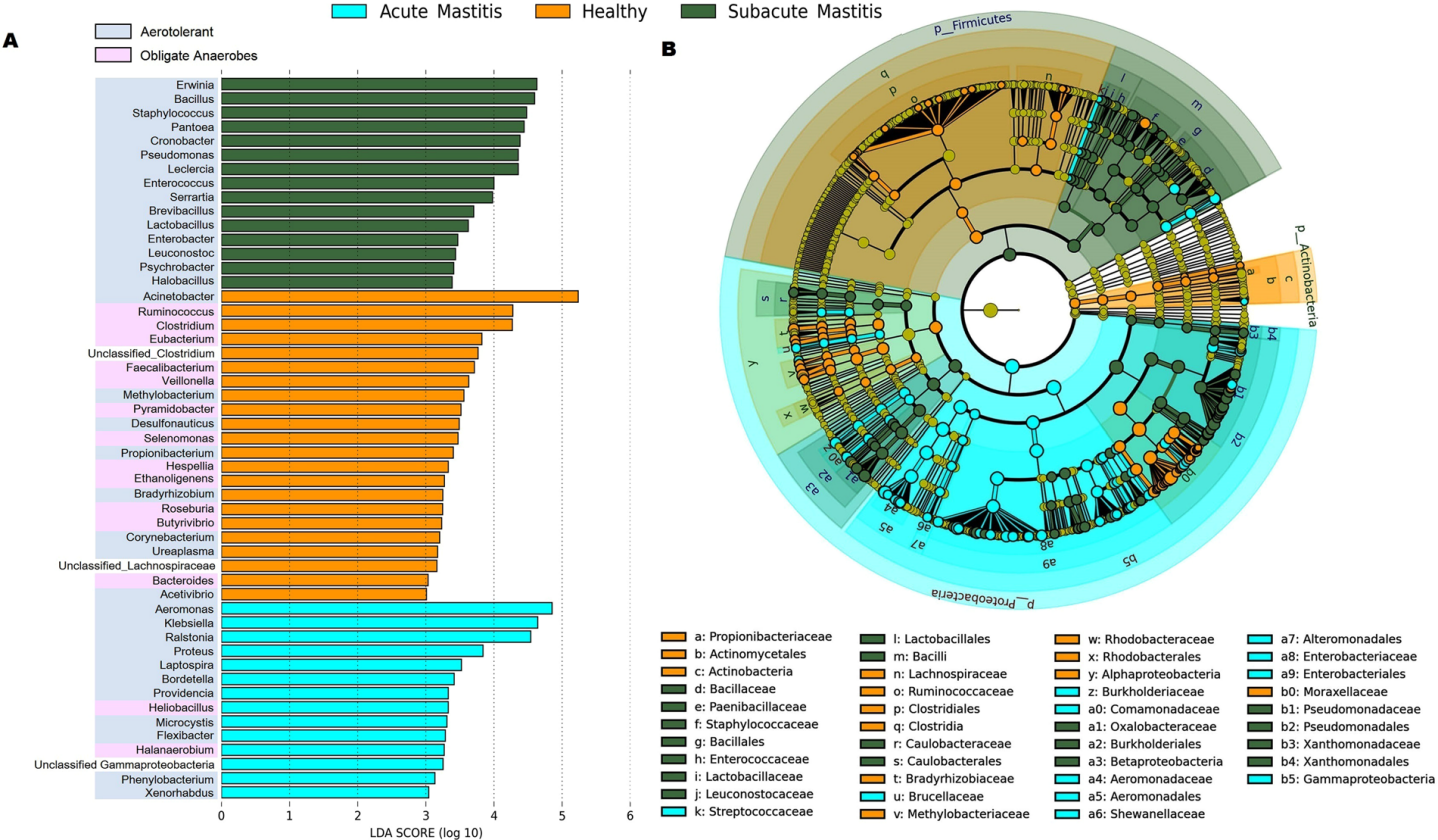
Differentially abundant bacterial taxa between healthy-controls, SAM and AM samples. (**A**) LEfSe was used to compare abundance of all bacterial genera between healthy-controls, SAM and AM samples. Significant bacterial genera were determined by Kruskal-Wallis test (P < 0.05) with LDA score greater than 3. (**B**) Cladogram representation of differentially abundant bacterial families detected using LEfSe. Different colors indicate the group in which clade was most abundant.

We next used LefSe to perform differentially abundant analysis at family and genus level between three groups of individuals. To analyze more accurately, we have only compared 197 genus that were present in at least 50% of samples in individual group. LEfSe analysis identified 51 differentially abundant genus in healthy, AM and SAM samples (Fig. 3A; alpha value for Kruskal Wallis and Wilcoxon test was set to 0.05 and LDA score > 3). Bacteria of the genus *Erwinia*, *Bacillus*, *Staphylococcus*, *Pantoea*, *Cronobacter* and *Pseudomonas* were more enriched in SAM samples (P < 0.05). While acute cases of mastitis harbored significantly more relative *Aeromonas*, *Klebsiella*, *Ralstonia*, *Proteus* and *Leptospira* (P < 0.05). Moreover, Cladogram that represents differentially abundant microbiota from phylum to family level is shown in Fig. 3B. At Family level, abundance of *Aeromonadaceae* (15.97%), *Burkholderiaceae* (7.65%), *Brucellaceae* (0.1%) and *Streptococcaceae* (0.030%) was significantly higher (P < 0.05) in samples from AM. While, *Enterobacteriaceae* (23.08%), *Bacillaceae* (7.44%), *Staphylococcaceae* (7.42%), *Pseudomonadaceae* (5.21%) and *Enterococcaceae* (1.93%) were the most prominent families found in SAM samples (Supplementary Fig. 3).

### Bacteria dysbiosis associated with positive aerotolerant odds ratio

We subsequently determined oxygen tolerance capacity of significantly enriched genera (LefSe; alpha value for Kruskal Wallis and Wilcoxon test was set to 0.05 and LDA score > 2) in healthy and mastitis individuals. Positive aerotolerant odds ratio (AOR 8.00, 95% Confidence Interval 3.07–20.92, P < 0.0001) suggested relative enrichment of aerotolerant organisms and depletion of obligate anaerobes in mastitis cases (Supplementary Fig. 4). Obligate anaerobes such as *Ruminococcus*, *Clostridium*, *Eubacterium*, *Faecalibacterium*, *Veillonella*, *Pyramidobacter*, *Selenomonas*, *Hespellia*, *Ethanoligenens*, *Roseburia* and *Butyrivibrio* drastically depleted in mastitis cases (Fig. 3A). Interestingly, with the exception of *Heliobacillus* and *Halanaerobium*, most of the genera enriched in SAM and AM were aerotolerant bacteria. Thus, bacterial dysbiosis in mammary gland during mastitis was associated with depletion of obligate anaerobes and enrichment of aerotolerant organisms.

### Enrichment of pathogenic species and depletion of beneficial commensals during mastitis

**Figure 4:**
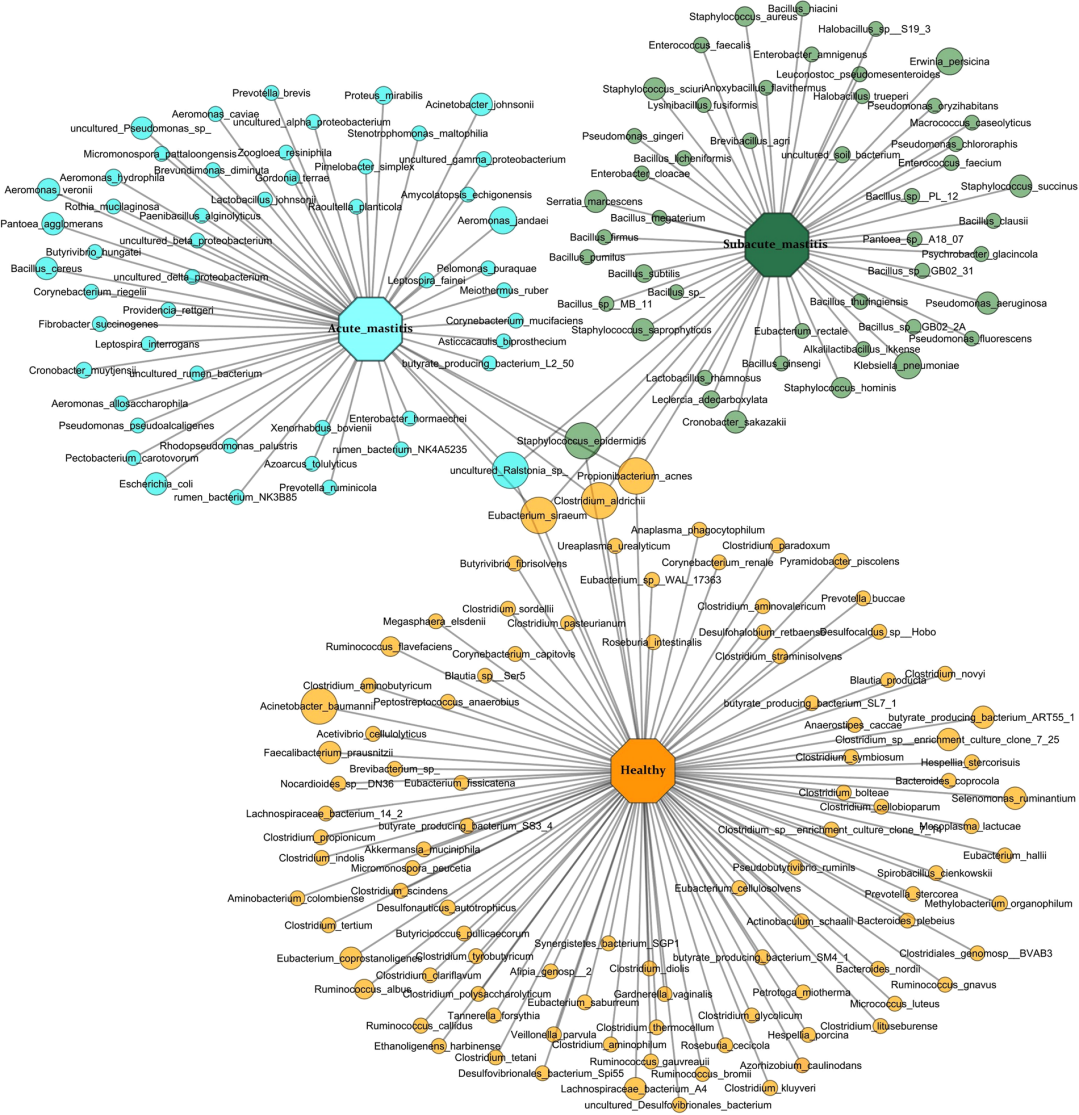
Network of bacterial species enriched in healthy-controls, SAM and AM individuals. Species core in half of the individual tested in each group were compared by LEfSe analysis. Cytoscape was used to construct network of enriched species in healthy-controls, SAM and AM samples.

We investigated next whether any specific species is associated with disease condition in SAM and AM cases. We detected a total of 2285 known species across all the individuals tested. At species level, very few were represented in all subjects; while most species were shared by limited number of individuals. To analyze more accurately, we only considered those species that were detected in 50% of samples in individual group. Such stringent condition resulted in fewer retained species, but more biologically meaningful interpretation. We detected 249 species in > 50% of healthy-controls, 170 species in > 50% SAM cases and 269 species in > 50% of AM cases. However, we detected only five species (*Staphylococcus epidermidis*, uncultured *Ralstonia sp*, *Propionibacterium acnes*, *Eubacterium siraeum* and *Clostridium aldrichii*) in all the individuals tested, suggesting highly variable species level community across all the samples. LEfSe analysis showed that 195 species (out of 424 species) were differentially abundant in three groups, including 97 species in healthy-controls, 47 species in SAM and 51 species in AM (Fig. 4; alpha value for Kruskal Wallis and Wilcoxon test was set to 0.05 and LDA score > 3).

Our data demonstrated that, *Acinetobacter baumannii* dominated the microbiota of healthy individuals, accounting for 36.27% of the healthy bacterial sequences. Other commonly found species in healthy human milk included *Ruminococcus flavefaciens*, *Propionibacterium acnes*, *Eubacterium siraeum*, *Akkermansia muciniphila*, *Ruminococcus albus*, *Faecalibacterium prausnitzii*, *Eubacterium coprostanoligenes*, *Veillonella parvula* and *Acinetobacter junii*, each representing 1.92% to 0.72% of healthy bacterial sequences. However, opportunistic pathogens such as *Erwinia persicina*, *Klebsiella pneumoniae*, *Cronobacter sakazakii*, *Leclercia adecarboxylata*, *Serratia marcescens*, *Bacillus subtilis*, *Enterococcus faecalis* and *Pseudomonas aeruginosa* were significantly enriched in SAM cases (P < 0.05). In addition to this, *S*. *saprophyticus* (*2*.*05%*), *S*. *epidermidis* (1.77%), *S*. *haemolyticus* (1.39%), *S*. *sciuri* (1.07%) and *S*. *aureus* (0.12%) of the genus *Staphylococcus* were differentially abundant in SAM compared to AM (P < 0.05). In contrast, AM harbored relatively more *Aeromonas jundaei*, *Pantoea agglomerans*, *Bacillus cereus*, *Escherichia coli*, *Aeromonas hydrophila*, *Acinetobacter johnsonii*, *Leptospira interrogans*, *Leptospira fainei*, uncultured *Ralstonia sp*. *and* uncultured *Pseudomonas sp*. in their breast milk samples (P < 0.05; Fig. 4).

### Predicted functional microbiota in healthy-controls and mastitis

**Figure 5:**
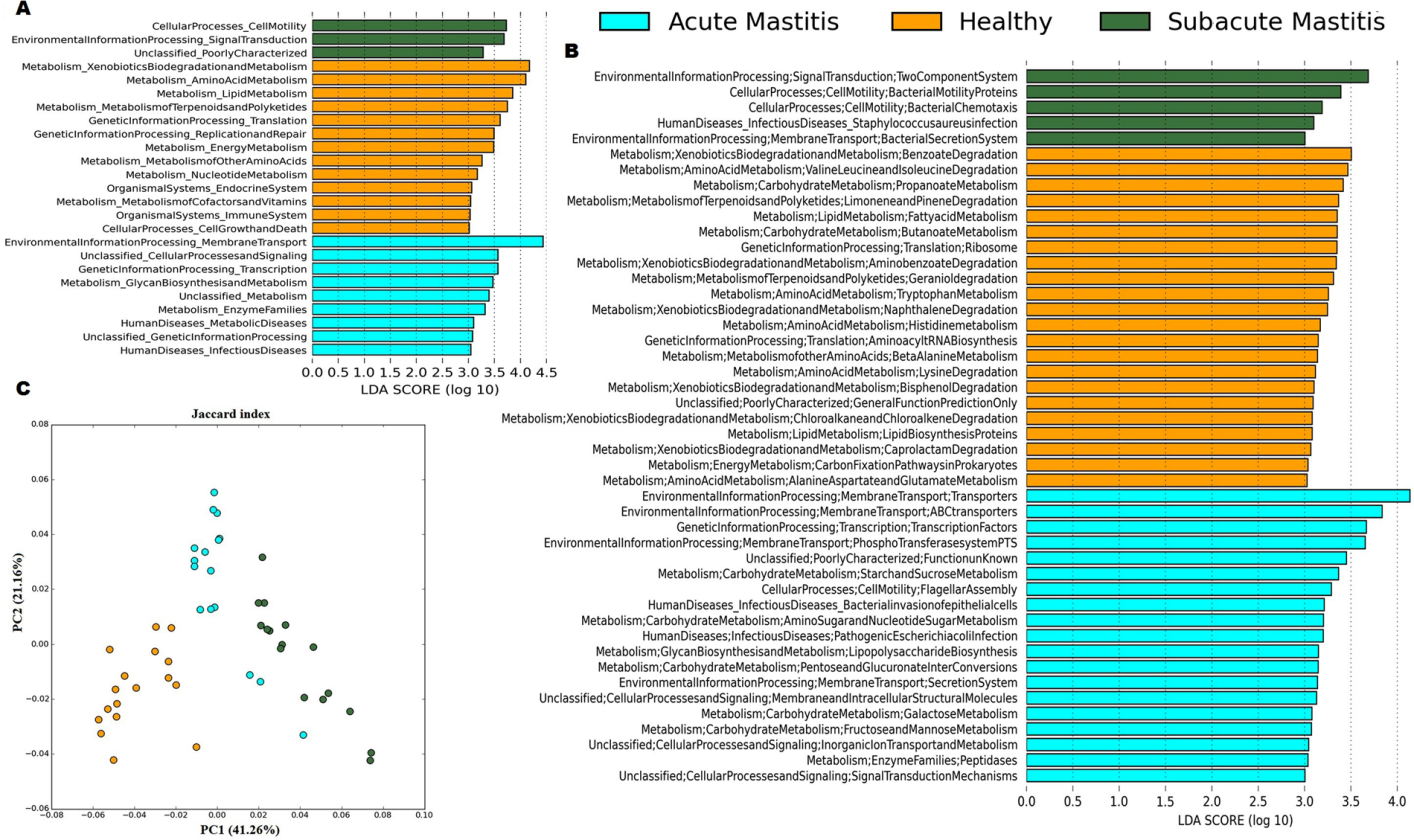
Predicted function of breast milk microbiota between healthy-controls, SAM and AM samples. LEfSe was used to compare abundance of predicted KEGG pathways at (**A**) level 2 and (**B**) level 3. The significant pathways were selected by Kruskal-Wallis test (P < 0.05) and LDA score of greater than 2.0. (**C**) PCoA of KOs based on Jaccard index revealed different clustering of healthy-controls from that of SAM and AM samples.

In addition to taxonomic profiling, we determined the functional gene component involved in dysbiosis of mammary gland during mastitis by computational method, PICRUSt (Phylogenetic Investigation of Communities by Reconstruction of Unobserved States). PICRUSt infers functional contribution of observed microbiota in individual samples based on raw 16 S rRNA marker gene count. At level 1, approximately 45% of genes belongs to metabolism, 18% of genes belongs to environmental information and processing, 14% genes belongs to genetic information processing and 1% of genes belongs to human diseases. Although, human milk microbial composition was strikingly invariable in healthy, SAM and AM samples, metabolic pathways were relatively stable (Supplementary Fig. 5). Next, we analyzed Kyoto Encyclopedia of Genes and Genomes (KEGG) pathways annotated at level 2 and 3 using LEfSe. At level 2, from a total of 39 KEGG features, 25 were significantly different between healthy-controls, SAM and AM samples (Fig. 5A; alpha value for Kruskal Wallis and Wilcoxon test was set to 0.05; LDA score > 2; P value < 0.05). Whereas at level 3, from a total of 263 KEGG features, 46 were differentially abundant between healthy-controls, SAM and AM samples (Fig. 5B; alpha value for Kruskal Wallis and Wilcoxon test was set to 0.05; LDA score > 2; P value < 0.01). Several KEGG features related to basic metabolism, immune system, biosynthesis and degradation of amino acids, nucleotide and lipids and immune system (Supplementary Fig. 6a) were more prominent in healthy-controls. In contrast, genes related to infectious disease, metabolic disease, glycan biosynthesis and metabolism and poorly characterized features were overrepresented in SAM and AM (Supplementary Fig. 6b,c). When examined at level 3, genes related to two component system, bacterial chemotaxis, *Staphylococcus aureus* infection, bacterial motility proteins and bacterial secretion system, bacterial invasion of epithelial cells and pathogenic *Escherichia coli* infection were significantly enriched in SAM and AM cases, respectively. Additionally, PCoA of KEGG Orthologous (KOs) based on Jaccard index revealed distinct clustering of healthy-controls from that of SAM and AM samples (Fig. 5C).

## Discussion

Previous culture dependent and independent studies provided insights into human milk microbiome and established that human milk is a rich source of beneficial microbes that plays a key role in maternal and infant’s health^8–10, 12–14, 25^. With this in mind, breastfeeding should be given more importance, at least during first six months after the birth. Mastitis is one of the common but major cause of premature weaning among lactating women. But there are very few microbiological studies that provides perception about etiology of mastitis^15, 21–23^. Healthy human milk microbiome has been studied in relation to gestation time, birth mode, and infant gender^11^. However, till date only one study has reported microbial dysbiosis associated with human mastitis through metagenomic approach. Jimenez *et al*., 2015, in that study, explored microorganisms involved in SAM and AM women and compared them with healthy-controls through shotgun metagenomic approach^16^. In this paper, for the first time, we investigated influence of SAM and AM on human milk microbial community through amplicon sequencing approach and compared them with healthy individuals.

Bacterial diversity is closely associated with disease condition. Earlier studies related to inflammatory bowel disease^26^, rheumatoid arthritis^27^, colorectal cancer^28^, liver cirrhosis^29^ and type 2 diabetes^30^ reported decreased species richness among subjects suffering with disease. In our study, alpha and beta diversity measures provided comprehensive picture of microbial dysbiosis associated with mastitis. Consistent with the previous study^16^, we observed decreased microbial diversity and species richness in SAM and AM samples in comparison to healthy-controls. Beta diversity accessed by unweighted and weighted UniFrac matrix revealed considerable microbial variation between healthy-controls, SAM and AM individuals. Moreover, we observed that microbiome of SAM and AM subjects fluctuates more than those of healthy individuals. While breast milk microbial composition was dominated by three main phylum: *Proteobacteria*, *Firmicutes* and *Actinobacteria*; their relative abundance varies between healthy, SAM and AM individuals. These results are in contrast to previous human milk microbiome studies^13, 31^, where *Firmicutes* was the most abundant phyla, but in agreement with recently reported metagenomic study^8, 9, 12^. Moreover, presence of uncultured bacteria at phylum level was also comparable to that of human mastitis microbiome study^16^, where 24% of reads were classified as uncultured bacteria.

The important feature of the dysbiosis associated with mastitis milk microbiota was significant depletion of normal commensal bacteria and increased abundance of opportunistic pathogens (pathobionts), already present in the human milk. Opportunistic pathogens are normal member of individual’s microflora, but become invasive and pathogenic and outgrew in numbers, when host is in unnatural condition or immune compromised^32^. SAM and AM individuals had higher abundance of *Proteobacteria* and lower abundance of *Firmicutes*, when compared to healthy-controls. Numerous families belonging to *Proteobacteria* phyla enriched in SAM and AM samples, including *Enterobacteriaceae*, *Aeromonadaceae*, *Burkholderiaceae* and *Pseudomonadaceae*. *Enterobacteriaceae* includes large number of opportunistic pathogens and its higher prevalence has been reported in several intestinal inflammatory diseases^33^, infectious diarrhea^34^ and multiple sclerosis patients^35^. SAM and AM microbiome was characterized by depletion of obligate anaerobes, including *Ruminococcus*, *Clostridium*, *Eubacterium*, *Roseburia*, *Clostridium*, *Butyrivibrio*, *Selenomonas* and *Faecalibacterium* and enrichment of several aerotolerant and opportunistic pathogens. Depletion of obligate anaerobes and enrichment of aerotolerant bacteria was also observed in complex microbiome of HIV infected patients^36^ and malnourished children^37^. Opportunistic aerotolerant bacteria, including *Pseudomonas*, *Staphylococcus*, *Ralstonia*, *Klebsiella* and *Pantoea* were also detected in previous healthy human milk studies^14, 38^, but in lower abundance. Previous studies dealing with culture dependent analysis of mastitis samples also identified *Staphylococcus*, *Pseudomonas*, *Klebsiella* and *Enterococcus* as major mastitis causing pathogens^22^. *Staphylococcus*, in particular, *S*. *aureus* was traditionally considered as major mastitis causing pathogen^15, 16, 21^. In our study, abundance of *Staphylococcus* was considerably higher in SAM and AM cases compared to healthy-controls.

Detection of *Acinetobacter*, as one of the most abundant genus in healthy milk is consistent with previous studies^8, 31, 39, 40^. In Chinese study, however, it was reported that abundance of *Acinetobacter* was higher in breast milk collected with standard collection procedure (without aseptic cleansing and rejection of foremilk), therefore it represents actual bacterial composition of milk ingested by infant^39^. Of note, in our study breast milk samples were collected following aseptic cleansing and rejection of foremilk samples. So, we find it unlikely that aseptic cleansing and rejection of foremilk can be a reason for lower number of *Acinetobacter* in their study. More studies are required to confirm the role of *Acinetobacter* in human breast milk. Moreover, nowadays there is great concern to include blank-control in metagenomic study but low microbial biomass and difficulty in amplifying bacterial sequences by PCR has assured us that our results reflects actual human milk microbial composition.

When examined at species level, we observed similar pattern of dysbiosis, enrichment of opportunistic pathogens and depletion of beneficial commensals in mastitis samples. However, these results should be interpreted with caution because none of the 16 S pipeline performed satisfactory at species level identification^24, 41^. SAM and AM microbiome contained significant lower abundance of beneficial bacteria, as previously reported^16^, including *A*. *muciniphila*, *F*. *prausnitzii*, *R*. *intestinalis* and *R*. *flavefaciens*. Both *A*. *muciniphila* and *F*. *prausnitzii* has been reported previously as anti-inflammatory commensals and negatively correlated with onset of inflammation^42, 43^. SAM and AM microbiome was associated with enrichment of known human opportunistic pathogens, including *S*. *aureus*, *S*. *epidermidis*, *S*. *hominis*, *K*. *Pneumoniae*, *S*. *marcescens*, *P*. *aeruginosa*, *E*. *faecalis* and *B*. *subtilis*. *B*. *cereus* and *E*. *coli*, suggesting their crucial role in mastitis pathogenesis.

The present study also revealed several important, predicted functional pathways that altered in abundance between healthy-controls, SAM and AM women. Metabolic pathways involved in bacterial colonization and proliferation, such as bacterial secretion system, two component system, *S*. *aureus* infection, bacterial chemotaxis and bacterial motility protein, pathogenic *E*. *coli*, infection were overrepresented in AM and SAM, respectively. Bacterial pathogens utilize bacterial secretion system^44^ and two component system^45^ to regulate virulence and its pathogenicity, through which they attack and infect host cell. These results, together with shift in microbial composition, suggest that different metabolic pathways observed in mastitis individuals were mainly due to altered bacterial communities.

In conclusion, our study confirmed complex microbial diversity, with significantly altered bacterial communities and imputed functions in SAM and AM women. We demonstrated that microbial dysbiosis associated with mastitis resulted into depletion of beneficial obligate anaerobes and enrichment of aerotolerant opportunistic pathogens. Several imputed metagenomic pathways differed between healthy-controls, SAM and AM, possibly reflecting metabolic changes associated with mastitis pathogenesis. However, future studies are needed to confirm the link between mastitis dysbiosis and depletion of obligate anaerobes.

## Material and Methods

### Participation and sample collection

Thirty two women, aged between 22–30 year, with acute (pain, redness and swelling on breast) and subacute symptoms (engorgement of breast and fever) of mastitis participated in the study and had given their informed consent. The present study was conducted according to the ethical guidelines of 1975 Declaration of Helsinki and sample collection procedure was approved by ethical committee of Govindbhai Jorabhai Patel Ayurveda College and Surajben Govindbhai Patel Ayurveda hospital (Approval No- IEC-3/GJPIASR/2015-16/E/3). Lactational consultant attending different Primary Health Care (PHC) and Community Health Care (CHC) centers of Valsad and Vadodara region of Gujarat, India diagnosed them with mastitis. Eighteen age matched healthy breast milk samples were also collected as a control. All the samples were collected between 12 and 30 days postpartum (Supplementary Table 1). None of the women had administered antibiotics or probiotics prior to the sample collection.

Approximately 4–5 ml of milk, expressed manually using sterile gloves from each breast, was collected in sterile falcon tubes. Before this, both hands of volunteers were washed with disinfectant hand soap. Nipples and surrounding areola were cleaned with cotton soaked with 70% ethyl alcohol. Subsequently, few drops of milk were discarded to avoid skin bacterial contamination and sample was collected. Samples were refrigerated at 4 °C, transported to laboratory on ice and frozen at −20 °C for subsequent DNA extraction.

### DNA extraction and sequencing

Thawed milk samples were mixed thoroughly and centrifuged at 16000 × g for 2 minutes. Supernatant was discarded and genomic DNA was isolated from remaining pellet using QIAamp Stool DNA fast mini kit with some modification. Quantity and purity of extracted DNA was checked on Nanodrop drop ND-1000 UV- Spectrophotometer (NanoDrop Technologies).

The V2-V3 hypervariable regions of 16S rRNA gene was amplified using fusion bacterial primer containing 101 F (5′ACTGGCGGACGGGTGAGTAA 3′) and 518 R (5′CGTATTACCGCGGCTGCTGG 3′) universal primer. Fusion primer contained titanium Lib-L adaptor sequences, key sequence followed by 10 bp of Multiple Identifier (MID) sequences unique for each individual samples. Each Polymerase Chain Reaction (PCR) consisted of 12.5 µl of KAPA HiFi HotStart ReadyMix (2X), 5 µl of each forward and reverse primer and 2.5 µl of 20ng/µl of template DNA. PCR was performed by initial denaturation at 95 °C for 3 min, followed by 25 cycles of denaturation at 98 °C for 20 s, annealing at 60 °C for 10 s, and extension at 72 °C 12 s; with final extension at 72 °C for 40 s. Each PCR reaction was kept in triplicate and pooled before purification.

PCR products were purified by QIAquick gel extraction kit (QIAGEN) followed by further purification with AMPure XP beads. After confirmation of library size and concentration with Agilent Bioanalyzer high sensitivity chip (Agilent, USA) and qubit fluorometer (Invitrogen, USA), libraries were diluted and equimolar pooled. Emulsion PCR was carried out using Ion one touchTM 400 kit (Life Technologies, USA) and sequencing of the clonal libraries were carried out on 318 chip using the Ion Torrent Personal Genome Machine (PGM) employing Ion Sequencing 400 kit (Life Technologies, USA) according to the manufacturer’s instruction.

### Sequencing data processing

Sequencing data were analyzed using MG-RAST (v3.6)^46^ and QIIME^47^ (version 1.9.0) pipeline. We obtained a total of 3.44 million raw sequences. Initially sequences were demultiplexed in QIIME using split_libraries.py script bundle according to known barcode sequences using default parameter (mean qual mean < 25, read length shorter than 200 were discarded). Quality filtered reads were uploaded on MG-RAST server for taxonomical and phylogenetic profiling. Low quality reads were again trimmed in MG-RAST using SolexaQA with default parameter. MG-RAST annotations were made against Greengenes (Ribosomal Database Project) database with minimum e-value of 1E-5 and minimum identity of 80%. Processed high quality sequences were downloaded from MG-RAST server and used for OTU picking and alpha and bets diversity analysis. OTU picking was performed at 97% sequences similarity and taxonomy was assigned by comparing representative sequences against Greengenes 13_8 reference database^48^ using RDP classifier^49^. To minimize the effect of uneven sequencing depth, each sample was rarefied to 4718 sequences per sample for subsequent analysis. One healthy sample was excluded from analysis due to low sequence number ( < 3000). Alpha and beta diversity analysis was performed by using several different metrics: observed OTU and Phylogenetic Diversity (PD) for within sample alpha diversity and unweighted UniFrac and weighted UniFrac PCoA for between sample beta diversity measurements. A non-parametric Kruskal-Wallis test was used to compare alpha diversity between healthy-controls, SAM and AM samples. Microbial compositional difference based on beta diversity was statistically evaluated using Adonis and ANOSIM test in QIIME (compare_categories.py). Network of species core in half of the individual tested in each group was constructed by Cytoscape (v.3.0.2)^50^.

### Imputed functional metagenomics using PICRUSt

We used PICRUSt to predict functional gene content of metagenomic samples based on raw 16S rRNA marker gene sequences^51^. For that, OTUs were picked by closed reference approach against Greengenes 13_8 reference database using pick_closed_reference_otus.py script bundle with QIIME. OTU table was then normalize based on copy number of 16S rRNA gene and metagenomes were predicted from the Kyoto Encyclopedia of Genes and Genomes (KEGG) catalogue.

### Determination of aerotolerant odds ratio

Bacterial oxygen tolerance database was downloaded (http://www.mediterranee-infection.com/article.php?laref=374) and according to their oxygen tolerance nature, each genera was assigned as ‘aerotolerant’, ‘obligate anaerobe’ and ‘unknown’. Aerotolerant odds ratio was calculated as described in^37^: AOR = (number of aerotolerant genera enriched in mastitis × number of obligate anaerobes depleted in mastitis)/(number of aerotolerant genera depleted in mastitis × number of obligate anaerobes enriched in mastitis). Positive aerotolerant odds ratio indicates enrichment of aerotolerant organisms and depletion of obligate anaerobes during mastitis. For calculation of aerotolerant odds ratio, we combined samples from SAM and AM.

### Statistical analysis

Unless otherwise mentioned in methods section above, all the statistical analysis were performed using STAMP^52^ and GraphPad Prism 5.1 software under the guidance of Statistician. Non-parametric Kruskal-Wallis test was used for multi-group comparison, while a non-parametric Mann-Whitney test was used for two group comparison. We used Linear Discriminate Analysis (LDA) effect size to identify differentially abundant taxa/ pathways between healthy, subacute and acute mastitis samples. LefSe uses a non-parametric Kruskal-Wallis test and unpaired Wilcoxon rank sum test to detect differentially abundant taxa among group of samples and then uses a LDA to estimate effect size of each taxa among particular groups. Differentially abundant KEGG pathways at level 2 and 3 were compared using LEfSe with default parameter (alpha value for Kruskal Wallis and Wilcoxon test was set to 0.05 and LDA > 2). Whereas, to identify differentially abundant taxa at all taxonomical level (from Phylum to Species level), LefSe analysis was carried out with one order of magnitude greater than default parameter (alpha value for Kruskal Wallis and Wilcoxon test was set to 0.05 and LDA > 3).

### Data availability

Data from this study have been deposited in the MG-RAST database under the following accession numbers: mgm4728095.30 - mgm4728134.3, mgm4728136.3, mgm4728139.3, and mgm4728696.3 - mgm4728702.3.

## Electronic supplementary material

Supplementary information
