# Supplementary material for: Culture independent assessment of human milk microbial community in lactational mastitis
Source: Sci Rep. 2017 Aug 10;7:7804. doi: 10.1038/s41598-017-08451-7 (PMC5552812; doi:10.1038/s41598-017-08451-7)
Supplement: Supplementary file 1 — Supplementary information [file 41598_2017_8451_MOESM1_ESM.pdf]

Culture independent assessment of human milk microbial community in lactational mastitis

Shriram H. Patel[1,3], Yati H. Vaidya[1], Reena J. Patel[1], Ramesh J. Pandit[2], Chaitanya G. Joshi[2], Anju P. Kunjadia[3*]

1 Ashok and Rita Patel Institute of Integrated Study and Research in Biotechnology and Allied Sciences, ADIT campus, New Vallabh Vidyanagar, Anand, Gujarat, India.

2 Department of Animal Biotechnology, College of Veterinary Science and A. H., Anand Agricultural University, Anand, Gujarat, India.

3 Center for Interdisciplinary Studies in Science and Technology (CISST), Sardar Patel University, Vallabh Vidya Nagar, Gujarat, India.

* Corresponding author: Anju Kunjadia, Department of Biomedical Science, CISST, Sardar Patel University, Gujarat-388121, India
Email: anjukunjadia@gmail.com
Phone. No. 9426583535

Supplementary Figure legends:

Supplementary Fig. 1: PCoA of Phylum to species level taxa revealed more pronounced clustering of healthy, SAM and AM samples at Genus and species level.

Supplementary Fig. 2: Dendogram based on ward linkage clustering of OTU level taxa show different clustering of healthy, SAM and AM samples.

Supplementary Fig. 3: Heatmap representation of most abundant family level taxa in healthy, SAM and AM samples.

Supplementary Fig. 4: Aerotolerant odds ratio at 95% CI revealed depletion of obligate anaerobes during mastitis.

Supplementary Fig. 5: Relative proportion of different metabolic pathways at level 2 between healthy, SAM and AM samples.

Supplementary Fig. 6: Predicted KEGG functions such as (a) immune system (b) metabolic disease and (c) infectious disease in individual healthy, SAM and AM samples.

Supplimentary Table legends:

Supplimentary Table 1: Characteristic of the participants involved in the study.

Supplimentary Table 2: Summary of sequencing and alpha diversity indices.

Supplementary Fig. 1

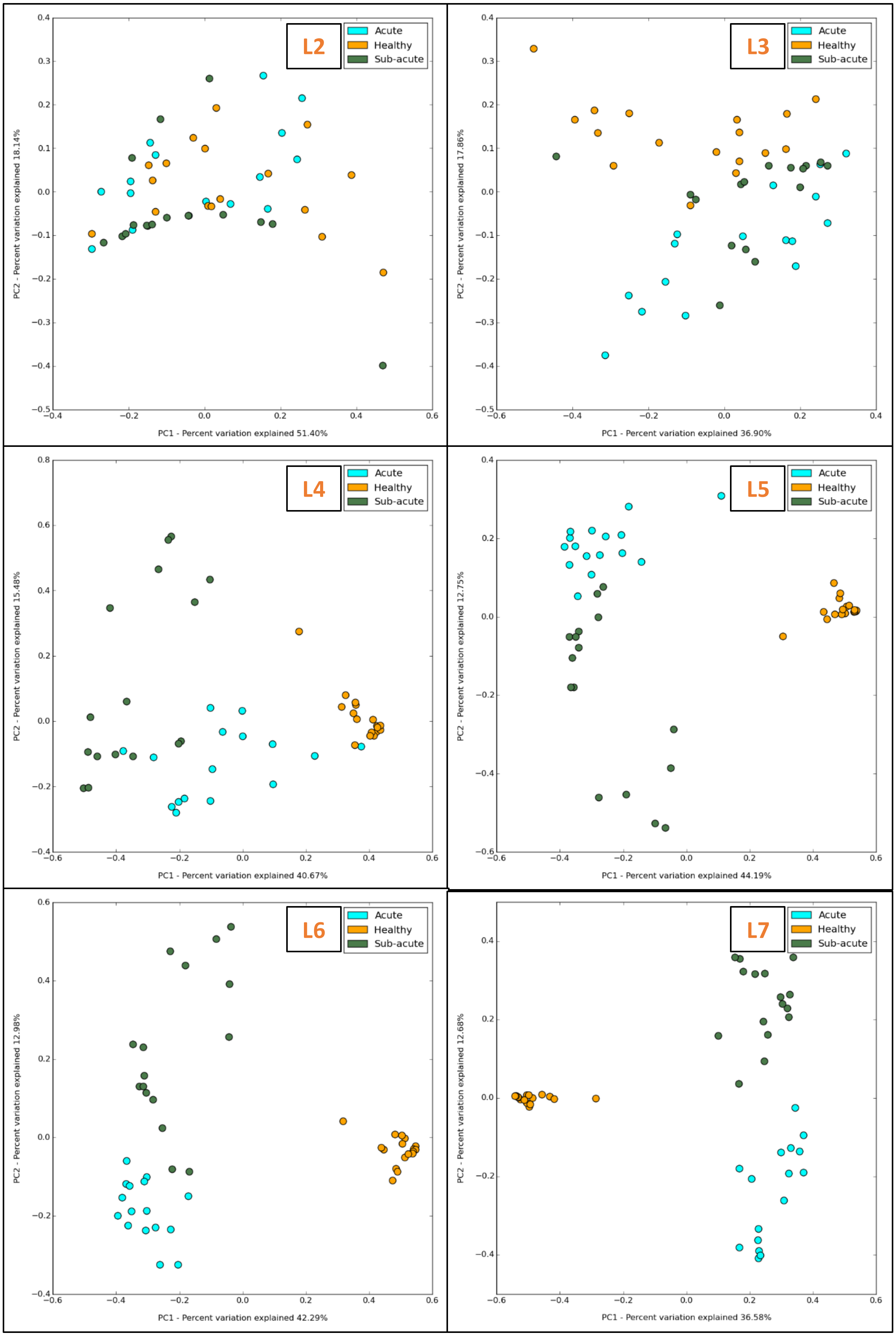

Supplementary Fig. 2

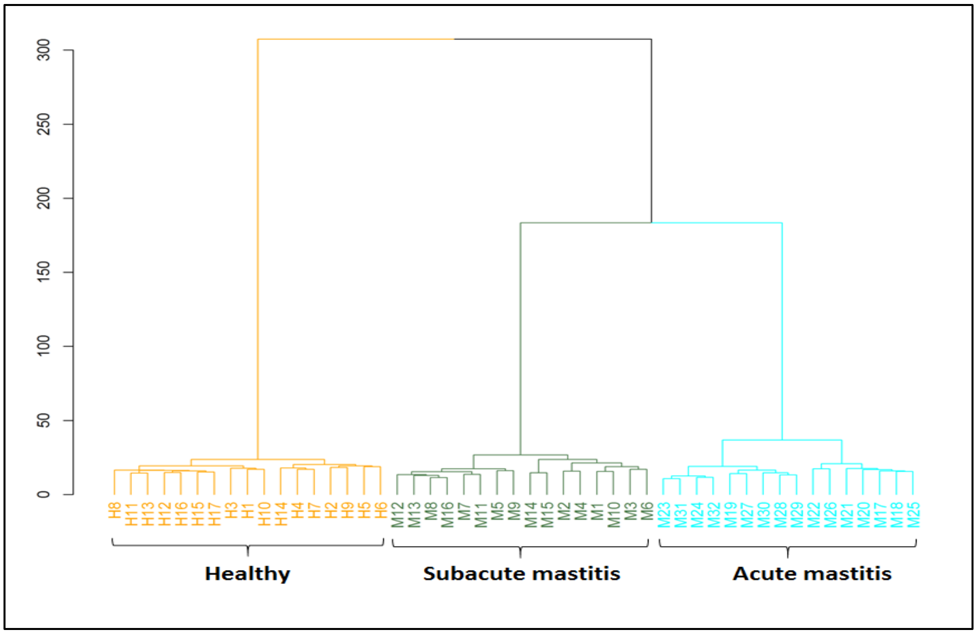

Supplementary Fig. 3

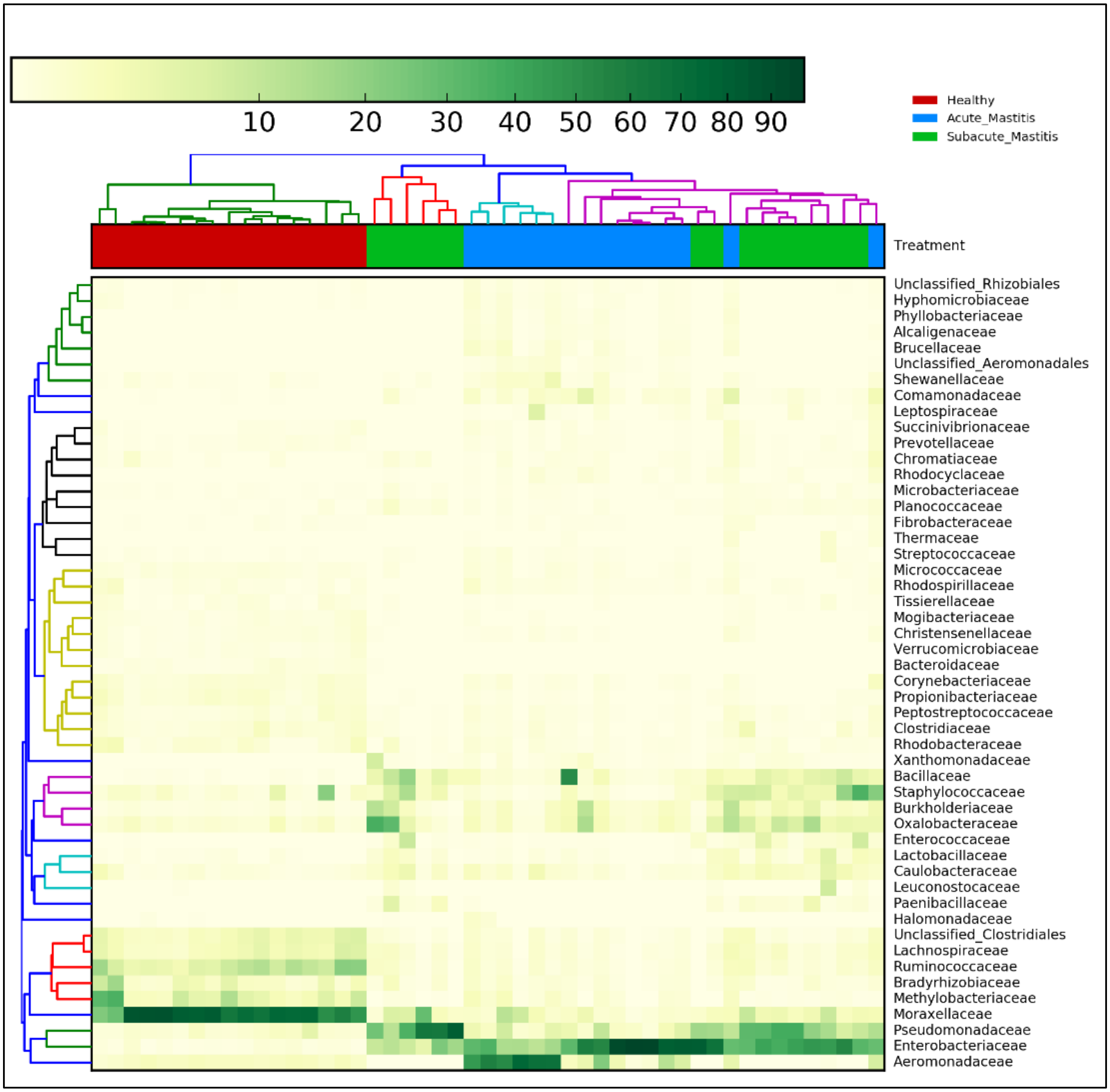

Supplementary Fig. 4

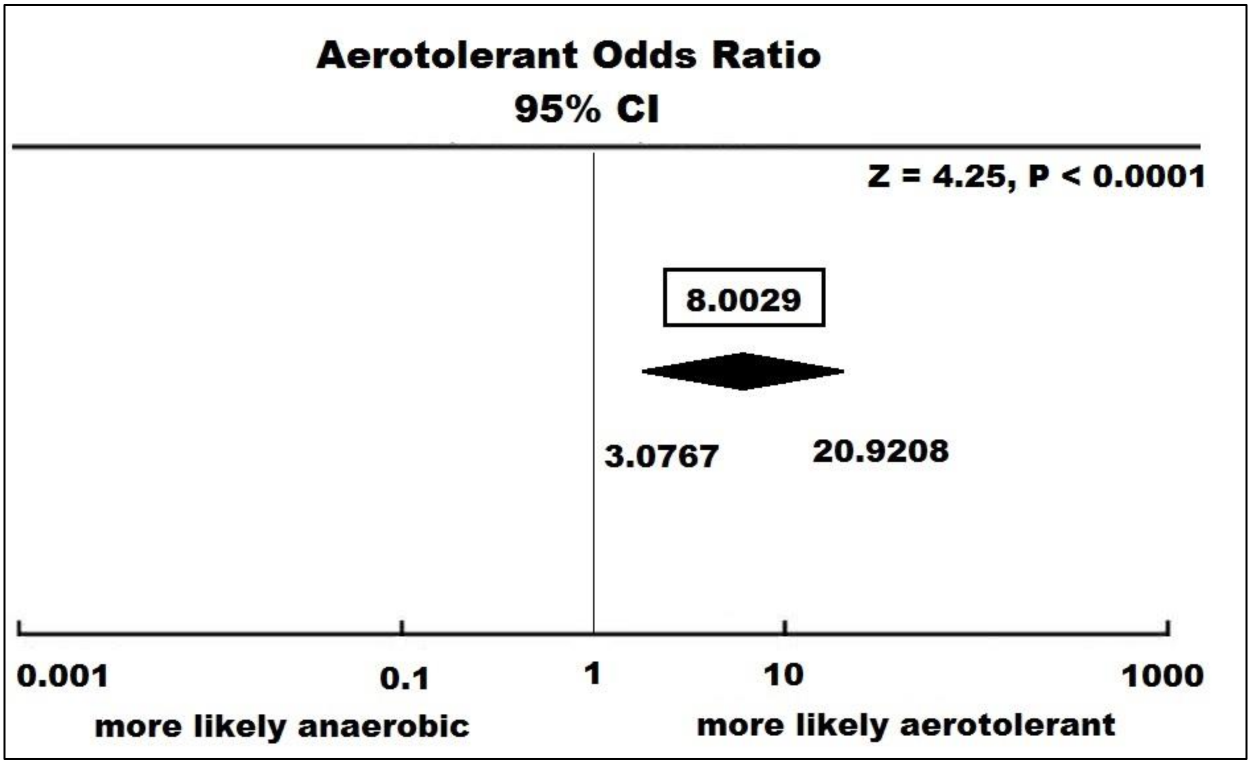

Supplementary Fig. 5:

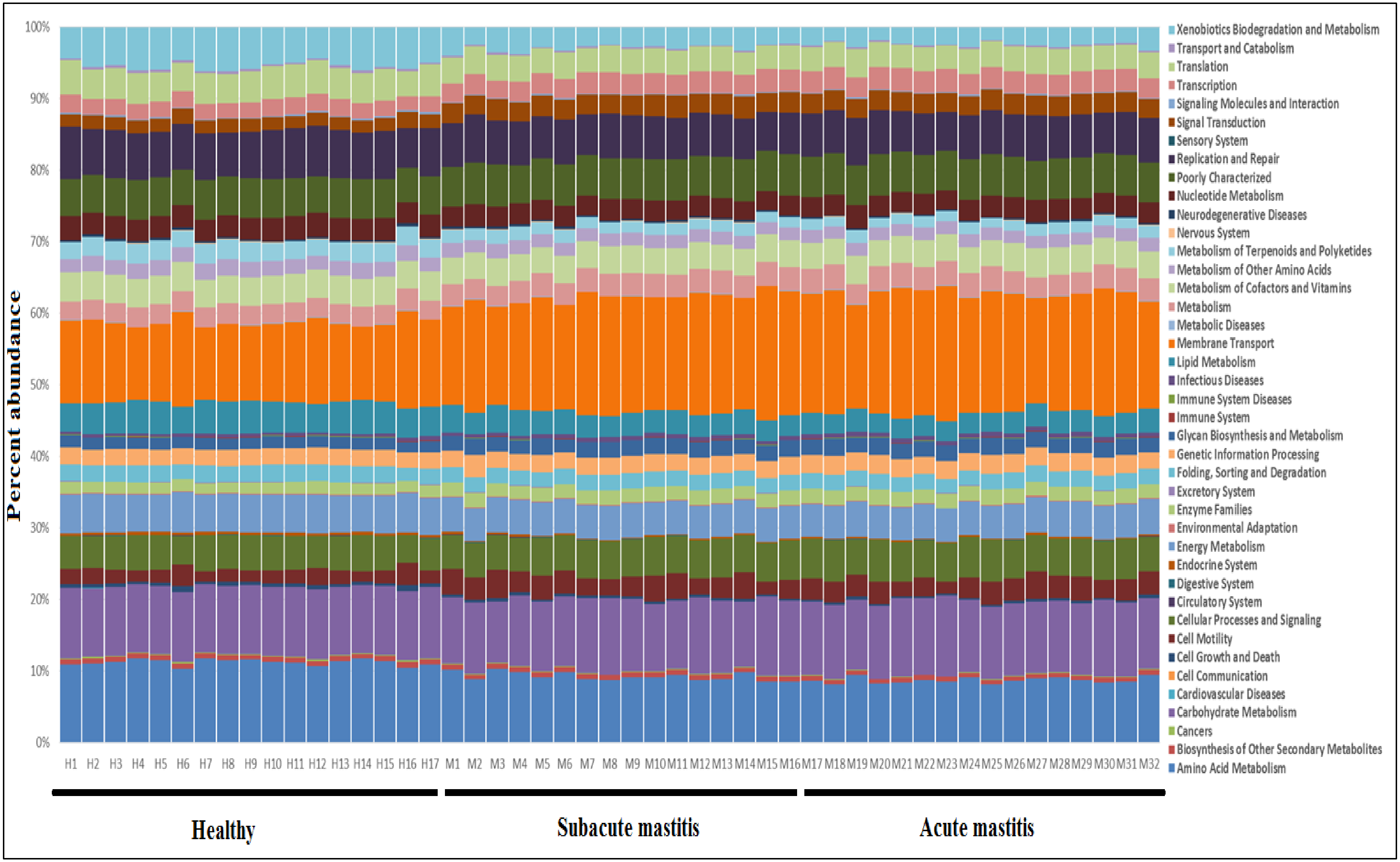

Supplementary Fig. 6

(a)

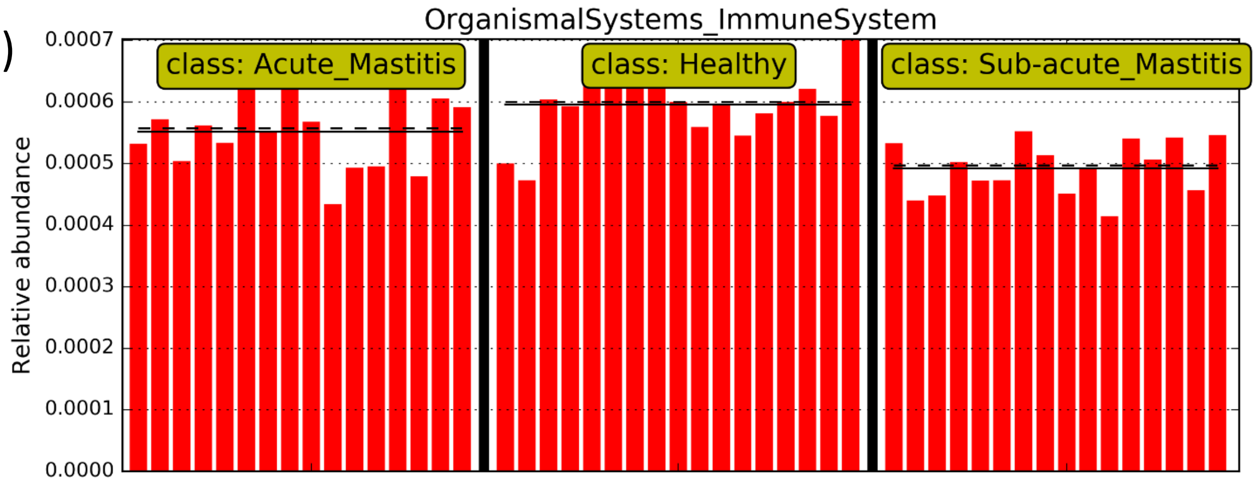

(b)

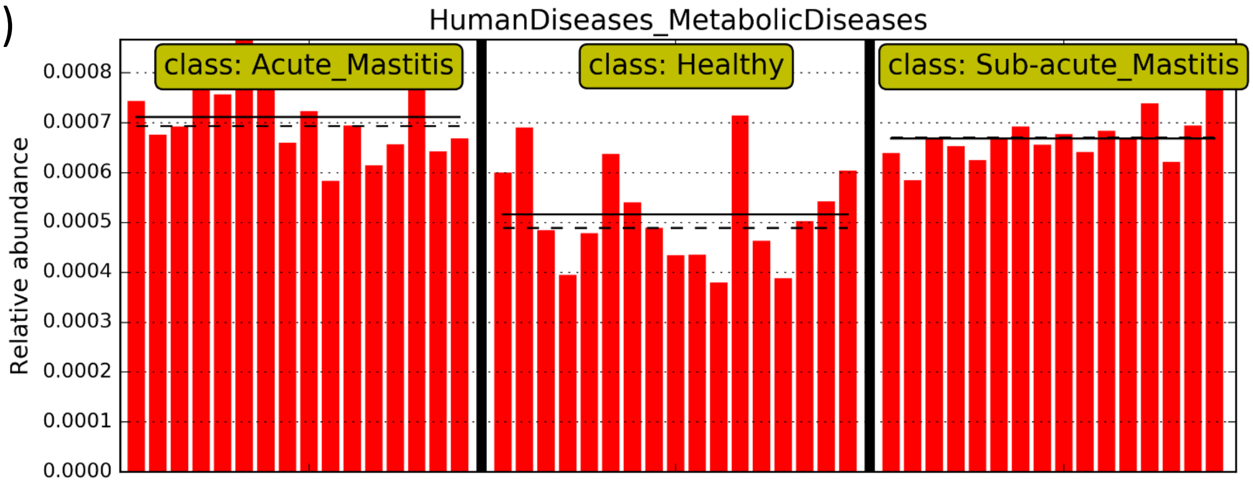

(c)

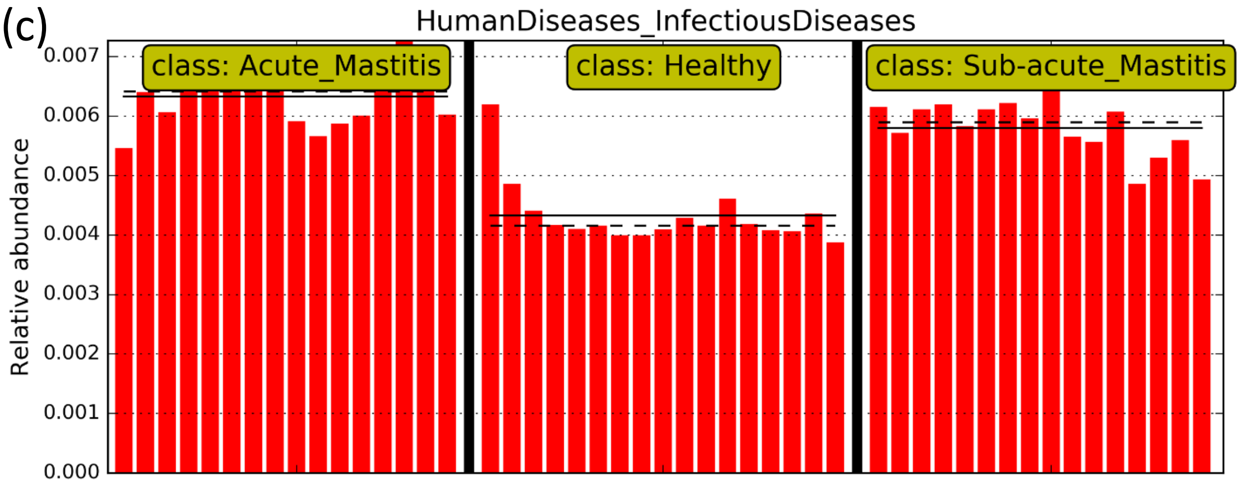

Supplementary Table 1:

	Healthy	Subacute mastitis	Acute mastitis
Study population, n	18	16	16
Age, years ± SD	25.29 ± 1.82	25.93 ± 2.37	26.12 ± 1.31
Postpartum period, days ± SD	20.58 ± 4.04	24.93 ± 3.92	24.75 ± 2.97

Supplementary Table 2:

Sample Group	Raw sequences	Quality Filtered Sequences	Observed OTUs*	Phylogenetic diversity*
Healthy	1066028	839140	506 (297-788)	20.37 (14.29-27.98)
Subacute mastitis	962855	647843	430 (255-603)	12.01 (5.24-19.61)
Acute mastitis	1416549	655902	427 (206-764)	16.38 (7.60-29.13)

***Asterisk indicate calculation of alpha diversity indices after rarefaction to 4718 sequences per sample.**
